# WNK4 is an essential effector of anterior formation in FGF signaling

**DOI:** 10.1111/gtc.12048

**Published:** 2013-03-21

**Authors:** Masahiro Shimizu, Toshiyasu Goto, Atsushi Sato, Hiroshi Shibuya

**Affiliations:** Department of Molecular Cell Biology, Medical Research Institute, Tokyo Medical and Dental University1-5-45 Yushima, Bunkyo-ku, Tokyo, 113-8510, Japan

## Abstract

With no lysine (K) (WNK) kinase family is conserved among many species and regulates SPAK/OSR1 and ion cotransporters. WNK is also involved in developmental and cellular processes, but the molecular mechanisms underlying its regulation in these processes remain unknown. In this study, we found that WNK4 is involved in fibroblast growth factor (FGF) signaling during *Xenopus* development. In *Xenopus* embryos, depletion of WNK4 by antisense morpholino oligonucleotides (MOs) results in a severe defect in anterior development and impaired expression of endogenous anterior markers. Defects in head formation or expression of anterior marker genes caused by suppression of endogenous WNK4 expression could be rescued by expression of wild-type WNK4, but not mutant WNK4 lacking its kinase activity. It is notable that morphants of *Xenopus* WNK4 inhibited the expression of anterior marker genes and the target genes induced by FGF signaling. Moreover, knockdown of Wnk4 significantly reduced the phosphorylation level of Osr1 induced by FGF. These results provide the first evidence that FGF signaling regulates WNK4 function required for anterior formation in *Xenopus* development.

## Introduction

With no lysine (K) (WNK) is a family of serine/threonine protein kinases that are characterized by an atypical location of the catalytic lysine and are conserved among many species, such as plants, nematode, fly, rat, mouse and human (Xu *et al*. 2000; Veríssimo & Jordan 2001; Moniz & Jordan 2010). There are four mammalian WNK family members, and positional cloning has identified two of them, WNK1 and WNK4, as genes linked to a hereditary form of human hypertension known as pseudohypoaldosteronism type II (PHAII) (Wilson *et al*. 2001). Several groups including our group previously discovered that WNK1 and WNK4 could phosphorylate and activate SPAK or OSR1 kinases, which in turn regulate various ion cotransporters, such as NKCC1, NKCC2 and NCC (Piechotta *et al*. 2002; Moriguchi *et al*. 2005; Vitari *et al*. 2005; Gagnon *et al*. 2006). Deletion in intron 1 of WNK1 gene in PHAII patients caused the increased expression of WNK1, which leads to hypertension by the misregulation of ion cotransporters (Wilson *et al*. 2001). WNK4 is known to be regulated by renin–angiotensin–aldosterone system. Angiotensin II induced WNK4 activation, which controlled NCC phosphorylation through SPAK/OSR1 kinases (San-Cristobal *et al*. 2009; Talati *et al*. 2010; van der Lubbe *et al*. 2011; Castañeda-Bueno *et al*. 2012). PHAII mutations in acidic region of WNK4 (D561A) disrupt this regulation (San-Cristobal *et al*. 2009). We also found that *WNK4*^*D561A/+*^ knock-in mice, model mice of human PHAII patient, caused phenotypes similar to those of PHAII (Yang *et al*. 2007). Furthermore, serum- and glucocorticoid-induced protein kinase (SGK1), which was activated by aldosterone, regulated WNK4 activity (Bhargava *et al*. 2001). The phosphorylation by SGK1 inhibited the function of inhibitory domain of WNK4 (Ring *et al*. 2007). PHAII mutations in C-terminal region of WNK4 (R1185C) lost the regulation by SGK1 (Na *et al*. 2013). These results suggest that the dysregulation of WNK4 contributes to the pathogenesis of hypertension in PHAII patients.

The WNK-SPAK/OSR1 pathway is known to regulate various ion cotransporters and is widely conserved among many species (Piechotta *et al*. 2002; Moriguchi *et al*. 2005; Vitari *et al*. 2005; Gagnon *et al*. 2006; Hisamoto *et al*. 2008; Sato & Shibuya 2013). *Wnk1* knockout mice die before embryonic day 13 (Zambrowicz *et al*. 2003; Sato & Shibuya 2013) and display defects in cardiac development (Xie *et al*. 2009). WNK1 is also required for cell division in cultured cells (Tu *et al*. 2011), and proliferation, migration and differentiation of neural progenitor cells (Sun *et al*. 2006). Furthermore, PHAII patients display a number of other clinical features, such as an intellectual impairment, dental abnormalities and impaired growth in addition to hypertension (Gordon 1986). Accordingly, the new role of the WNK signaling pathway described here may provide further insight into the development and pathogenesis of PHAII. Recently, we have identified Lhx8/Awh as a new downstream molecule in the WNK-SPAK/OSR1 pathway and discovered a novel function for the WNK-Lhx8 pathway in neural development (Sato & Shibuya 2013).

In vertebrate, neural induction is established by inhibition of bone morphogenetic protein (BMP) signal in the ectoderm (Bier 1997; Sasai & De Robertis 1997; Wilson & Hemmati-Brivanlou 1997; Weinstein & Hemmati-Brivanlou 1999). Several inhibitors of BMP such as noggin and chordin are isolated from organizer region in *Xenopus*. However, inhibitory signals from organizer are not enough for neural induction, and other signals including fibroblast growth factor (FGF) are necessary for neural induction (Launay *et al*. 1996; Hongo *et al*. 1999; Powers *et al*. 2000). For examples, a kinase inhibitor of FGF receptor 1 (FGFR1) inhibits down-regulation of BMP in chick explants (Wilson *et al*. 2000). In *Xenopus*, FGF signals contribute to neural induction in animal cap cells (Kengaku & Okamoto 1995; Lamb & Harland 1995), and over-expression of dominant negative forms of FGFRs, which lack the intracellular domain, inhibits neural induction by noggin and chordin (Launay *et al*. 1996; Sasai *et al*. 1996).

FGF ligands contain more than 20 family members (Powers *et al*. 2000; Fukumoto 2008). Four FGFR genes, *FGFR1* to *FGFR4*, are identified in vertebrates (Dailey *et al*. 2005; Eswarakumar *et al*. 2005). Each FGFR contains an FGF binding extracellular domain and a catalytic intracellular domain. When a ligand binds to a receptor, receptor dimerizes via the extracellular domain and its intracellular tyrosine kinase domain is autophosphorylated, causing its downstream signaling. The main downstream pathways include the mitogen-activated protein kinase (MAPK) pathway, the phosphatidylinositol-3 kinase (PI3K) pathway and the phospholipase Cγ (PLCγ) pathway (Dailey *et al*. 2005; Eswarakumar *et al*. 2005).

In the present study, we report the novel finding that *Xenopus* WNK4 (xWNK4) is involved in FGF signaling. Depletion of xWNK4 resulted in defects in anterior neural development in *Xenopus* embryos, including the loss of eye and head structures. The phenotypes induced by depletion of endogenous xWNK4 were rescued by over-expression of wild-type *xWNK4,* but not by a kinase negative mutant of *xWNK4*. Moreover, the expression of FGF-inducible genes was inhibited by the depletion of xWNK4. These results reveal a new role of WNK4 function during anterior formation in *Xenopus* embryos.

## Results

### Isolation of *Xenopus* WNK4 cDNA

**Figure 1:**
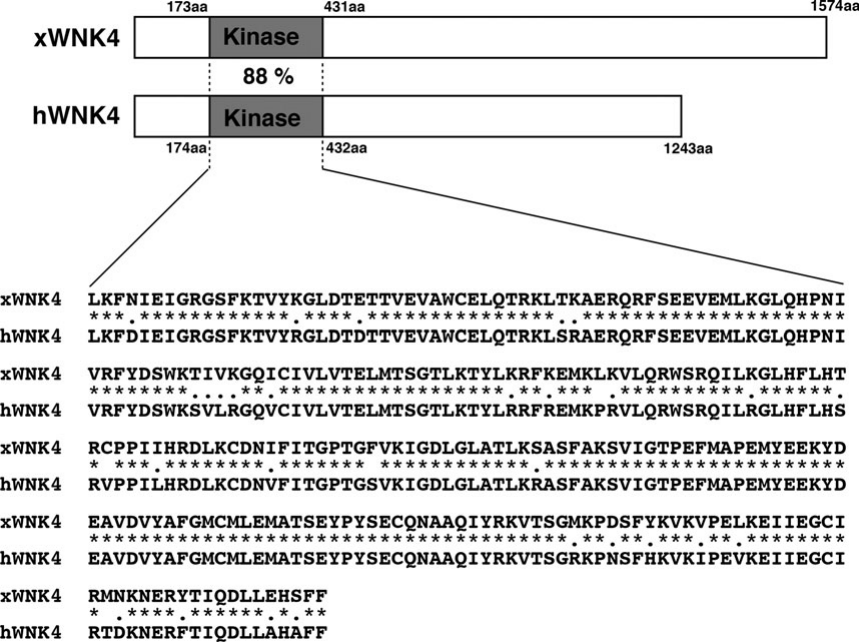
Alignment of the *Xenopus* WNK4 (xWNK4) and human WNK4 (hWNK4). The conserved amino acids are represented by asterisks (*). The kinase domains have high identity (88%) and are shaded gray.

To isolate *Xenopus WNK4* (*xWNK4*) cDNA, we searched the *Xenopus* expressed sequence tag database with high homology to human WNK4 (hWNK4) and isolated the full-length cDNA from a *Xenopus* tailbud cDNA library by PCR method using primers based on the predicted WNK4 coding sequence (see Experimental Procedures). We found a single open reading frame of 1574 amino acids as a *Xenopus* homologous gene. *Xenopus* WNK4 contains a single kinase domain near the N-terminal region, which has remarkable homology (88% identity) with the human WNK4 one (Fig. 1A). This suggests that xWNK4 also regulates the downstream kinases, such as SPAK/OSR1 (Moriguchi *et al*. 2005; Vitari *et al*. 2005; Gagnon *et al*. 2006).

### *Xenopus* WNK4 is expressed maternally and throughout early embryogenesis

**Figure 2:**
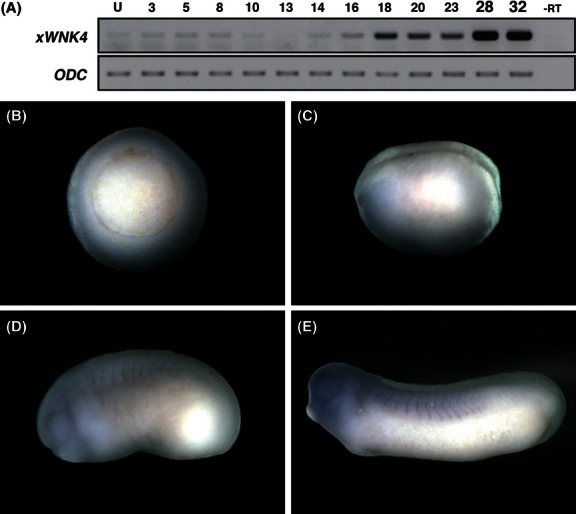
Temporal and spatial expression of *xWNK4*. (A) RT-PCR was carried out using 1 μg of total RNA extracted from *Xenopus* embryos at different stages. ‘U’ indicates the unfertilized eggs, and numbers indicate the developmental stages. *xWNK4* is maternally expressed, and its zygotic expression increased after neurula stage (st.14). (B–E) Localization of *xWNK4* transcripts by whole-mount *in situ* hybridization. At the gastrula stage, *xWNK4* is entirely expressed in ectodermal region (B, vegetal view, stage 10.5). Expression of *xWNK4* is gradually restricted to anterior neural region (lateral view, C; stage 17, D; stage 23, E; stage 27).

To assess the possible involvement of *xWNK4* for early development, we first examined the temporal and spatial expression patterns of *xWNK4* by RT-PCR analysis and whole-mount *in situ* hybridization. RT-PCR analysis of *xWNK4* expression in early *Xenopus* embryos revealed that *xWNK4* mRNA was expressed maternally, and its zygotic expression was increased after the neurula stage (Fig. 2A). Expression of *xWNK4* mRNA was detected entirely in ectoderm region at gastrula stage (Fig. 2B). After the neurula stage, transcripts of *xWNK4* were gradually restricted to neural region, especially in anterior neural regions involving eye and brain at later stages (Fig. 2C–E). These results suggest that xWNK4 might act in *Xenopus* early embryogenesis.

### *Xenopus* WNK4 functions in anterior neural development

**Figure 3:**
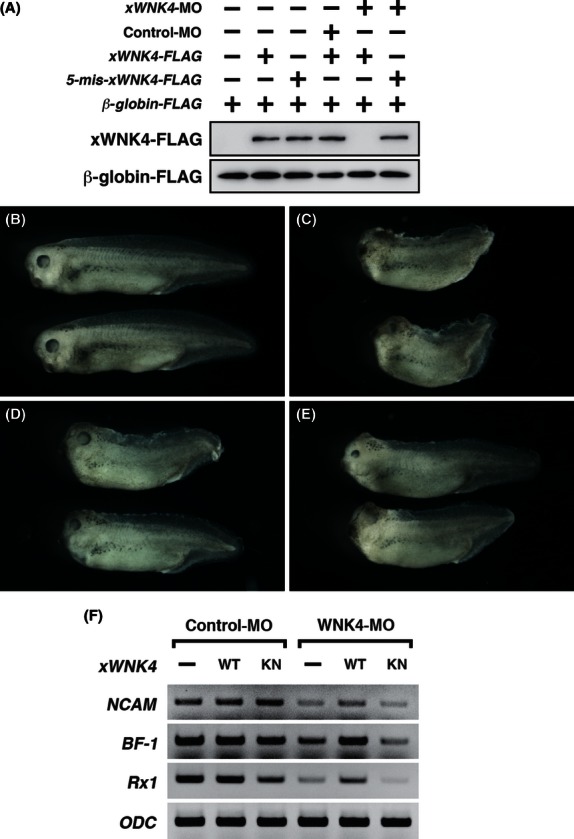
xWNK4 functions in anterior neural development. (A) Morpholino (MO) (20 ng) and FLAG-tagged mRNAs (1000 pg) were co-injected with β*-globin-FLAG* mRNA (200 pg) as loading control into the animal poles of 4-cell stage embryos, and the injected animal caps were dissected at stage 10. Lysates from the animal caps were subjected to Western blotting with anti-FLAG antibody (M2, Sigma). (B–E) Phenotypes of injected embryos at stage 35. Control-MO (30 ng) or *xWNK4*-MO (30 ng) was co-injected with *xWNK4* mRNA (1000 pg) or kinase negative *xWNK4* mRNA (1000 pg) into two dorso-animal blastomeres of 8-cell embryos. (B) Control-MO. (C) *xWNK4*-MO. (D) *xWNK4*-MO with *xWNK*4 mRNA. (E) *xWNK4*-MO with kinase negative *xWNK4* mRNA. (F) RT-PCR analysis of neural marker genes, *NCAM* (pan-neural), *BF-1* (forebrain) and *Rx1* (eye). RNAs from head regions of injected embryos were extracted at stage 25.

To examine the function of xWNK4 in early embryogenesis, we synthesized antisense morpholino oligonucleotide (MO) against *xWNK4* (See Experimental procedures). By Western blotting analysis, we confirmed that injection of the *xWNK4* – MO specifically reduced expression of xWNK4 protein (Fig. 3A). When *xWNK4*-MO was injected into two dorso-animal blastomeres at 8-cell stage, which develops mainly into neuroectodermal tissues and head structure, the resulting phenotype interfered with the head formation (Fig. 3B,C). We also confirmed that depletion of xWNK4 reduced expression of neural marker genes such as pan-neural marker, *NCAM*; forebrain marker, *BF-1*; eye marker, *Rx1* (Fig. 3F). The anterior defects and the reduction of anterior neural marker expression by *xWNK4*-MO were rescued by co-injection of wild-type *xWNK4* mRNA, but not by kinase-inactive *xWNK4* mRNA (Fig. 3D–F). These results suggest that xWNK4 is specifically involved in anterior formation in *Xenopus* embryos.

### *Xenopus* WNK4 is involved in FGF signaling

**Figure 4:**
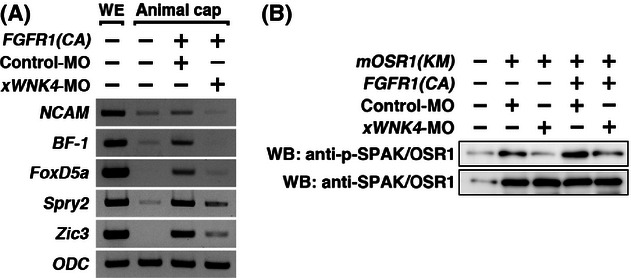
*Xenopus* WNK4 is involved in fibroblast growth factor (FGF) signaling. (A) RT-PCR analysis of neural marker genes, *NCAM* and *BF-1*, and FGF-target genes, *FoxD5a*, *Spry2* and *Zic3*. Control-MO (10 ng) or *xWNK*4-MO (10 ng) was co-injected with *FGFR1(CA)* mRNA (250 pg) into four animal blastomeres of 8-cell embryos, and animal cap explants were dissected at stage 9 and cultured until stage 18. RNAs from animal cap explants were extracted at stage 18. WE: whole embryos. (B) Western blotting analysis. Control-MO (30 ng) or *xWNK*4-MO (30 ng) was co-injected with *FGFR1*(*CA*) mRNA(250 pg) and/or *mOSR*1 mRNA (50 pg) into four animal blastomeres of 8-cell embryos. Animal caps were dissected at the early gastrula stage (st.10) and were lysed.

It is known that the FGF signaling pathway positively regulates *Xenopus* anterior neural development (Hongo *et al*. 1999). To examine whether xWNK4 would be involved in these signaling pathways in early embryogenesis, we analyzed the effects of *xWNK4*-MO on the expression of FGF-inducible neural marker genes in *Xenopus* animal cap cells. We found that co-injection of *constitutively active FGF receptor 1* (*FGFR1*(*CA*)) mRNA with *xWNK4*-MO decreased the expression of neural marker genes, *NCAM* and *BF-1*, relative to animal cap cells co-injected with the control morpholino (Fig. 4A). Moreover, the expression of FGF-target genes, *FoxD5a*, *Spry2* and *Zic3*, was also inhibited by depletion of endogenous xWNK4. We further attempted to examine the WNK-SPAK/OSR1 pathway in the FGF stimulation. A previous study has reported that WNKs are activated by hypertonic stimulation (Zagórska *et al*. 2007). To investigate whether WNK4 is activated by FGF stimulation, we carried out Western blotting analysis using antiphospho OSR1 antibody, which recognizes Ser325 of mouse OSR1 (mOSR1) phosphorylated by WNK kinases, with animal cap cells. We found that the phosphorylation level of mOSR1 was increased by the expression of *FGFR1(CA)* in animal cap cells (Fig. 4B). However, depletion of xWNK4 significantly reduced the phosphorylation level of mOsr1 (Fig. 4B). Taken together, these results indicate that xWNK4 may positively contribute to the FGF signaling pathway.

## Discussion

In this report, we found a novel role of WNK4 gene in *Xenopus* embryos. Injection of *xWNK4*-MO demonstrated that xWNK4 is involved in the anterior formation during early embryogenesis. Moreover, in animal cap cells, *xWNK4*-MO inhibits the anterior neural marker, and FGF-target genes induced by FGFR1(CA). These results suggest that xWNK4 contributes to the FGF signaling in *Xenopus* embryos.

WNK has been shown to interact directly with and to phosphorylate SPAK/OSR1, which in turn regulates various ion cotransporters, such as NKCC1, NKCC2 and NCC (Piechotta *et al*. 2002; Moriguchi *et al*. 2005; Vitari *et al*. 2005; Gagnon *et al*. 2006). Moreover, we have recently shown that WNK regulates the expression of *Lhx8* to play an essential role in neural development (Sato & Shibuya 2013). We also indicated the possibility that *Drosophila* WNK directly interacted with and phosphorylated Fray, fly homologue of SPAK/OSR1, which in turn regulated the expression of *Arrowhead*, fly homologue of *Lhx8*, and that this pathway is important for *Drosophila* abdominal and neural development (Sato & Shibuya 2013). Thus, WNK-SPAK/OSR1 pathway may be able to be involved in multiple function, and possibly, its activities are modulated through the phosphorylation of SPAK/OSR1 by WNK. Our results showing that FGFR1(CA) can activate mOSR1 in animal cap cells (Fig. 4B) suggest that WNK activity is also regulated by FGF. These observations lead us to hypothesize that WNK, presumably activated by FGF, may interact with and regulate the expression of FGF-induced target genes. Actually, *xWNK4*-MO inhibited the expression of FGF-induced target genes in animal cap cells. However, it has not been directly shown whether WNK-SPAK/OSR1 pathway regulates the target genes following stimulation of the FGF signaling pathway. Additional experiments will be required to test this hypothesis, and further molecular analysis will be needed to assess the role of WNK-SPAK/OSR1 pathway in FGF signaling.

To date, two regulatory pathways for head development have been proposed. One is a default state that is triggered by inhibiting growth factor signals required for trunk development, such as those induced by Wnt, BMP and Nodal (Harland 2000). The other includes growth factor-mediated active pathways, such as FGFs and IGFs (Hardcastle *et al*. 2000; Pera *et al*. 2001; Richard-Parpaillon *et al*. 2002). These pathways function in a parallel and/or cooperative manner to promote anteriorizing activity and to suppress the trunk mesoderm. Although the actual mediator(s) for anterior neural formation, which is regulated by WNK-SPAK/OSR1 pathway in FGF signaling, is unclear at present, it is known that WNK activates the expression of Lhx8/Arrowhead gene and is involved in neural differentiation of mammalian cells and *Drosophila* neural development (Sato & Shibuya 2013). It is possible that WNK activates the expression of *Lhx8* through the action of a yet-to-be-identified mediator(s) that regulates anterior tissue development, and further studies will be needed to identify the precise mediator(s) involved in this process. It will also be important to determine whether there is cross-talk between FGF-WNK-SPAK/OSR1 pathway and other signaling pathways to mediate anterior tissue development.

## Experimental procedures

### Plasmid construction and morpholino oligonucleotides

The *Xenopus WNK4 (xWNK4)* was amplified by PCR from a *Xenopus* tailbud cDNA library using the following primers: 5′-CGCCATCGATGGTACTTGTGTACATTGCTC-3′ and 5′-CGGCTCTAGATTACTTTACTTTTTCATCCTG-3′, and subcloned into pCS2+ vector. A kinase negative mutant of xWNK4 was constructed by replacing the lysine residue with methionine: K185M. A constitutive active FGFR1 was constructed by replacing the cysteine residue with tyrosine: C337Y. The MO (Gene Tools, LLC) used here were 5′-CCTCTTACCTCAGTTACAATTTATA-3′ (Control-MO), 5′-CAGGGACACTAACAGCAAACATCTC-3′ (*xWNK4*-MO). The specificity of *xWNK4*-MO was confirmed by its ability to inhibit the translation of FLAG-tagged mRNAs containing the targeted site with or without 5-mismatched sequences. MO (20 ng) and FLAG-tagged mRNAs (1000 pg) were co-injected with *β-globin-FLAG* mRNA (200 pg) as loading control into the animal poles of 4-cell stage embryos, and the injected animal caps were dissected at stage 10. Lysates from the animal caps were subjected to Western blotting with anti-FLAG antibody (M2, Sigma).

### Embryo handling

MOs and mRNAs were injected into four animal blastomeres at the 8-cell stage for dissection of animal caps or into two dorso-animal blastomeres at the 8-cell stage for RT-PCR analysis and observation of embryo phenotypes. Animal cap explants of the injected embryos were dissected at the late blastula stage (st.9), and total RNA was extracted at the neurula stage (st.18) for RT-PCR analysis. Head regions of embryos were dissected at the tailbud stage (st.25), and total RNA was extracted for RT-PCR analysis.

### RT-PCR analysis

Total RNA was prepared using TRIzol (Invitrogen). cDNA synthesis was carried out using Moloney murine leukemia virus reverse transcriptase (Invitrogen). The sequences of the primer pairs were as follows. *Ornithine decarboxylase* (*ODC*): Forward 5′-AAAATGGATGACTGCGAGATGGG-3′; Reverse 5′-AATGAAGATGCTGACTGGCAAAAC-3′. *xWNK4*: Forward 5′-ATGTGACCGTGTTGTTGAGTGCCAG-3′; Reverse 5′-GAGGAAGAGAATGACCTTGAGAGC-3′. *NCAM*: Forward 5′-CACAGTTCCACCAAATGC-3′; Reverse 5′-GGAATCAAGCGGTACAGA-3′. *BF-1*: Forward 5′-TCAACAGCCTAATGCCTGAAGC-3′; Reverse 5′-GCCGTCCACTTTCTTATCGTCG-3′. *Rx1*: Forward 5′-CCCCAACAGGAGCATTTAGAAGAC-3′; Reverse 5′-AGGGCACTCATGGCAGAAGGTT-3′. *FoxD5a*: Forward 5′-CCCACAAGAAACTGACTCTC-3′; Reverse 5′-CTGAGGTTGGATAGCACTGT-3′. *Spry2*: Forward 5′-CATAGCACAGGTGAACGGATGT-3′; Reverse 5′-AAAGTTCCAGAAGGCGAAGG-3′. *Zic3*: Forward 5′-CAGGCTTCTGGATATGCCAATT-3′; Reverse 5′-TGGTGAGCAGCTACATTCATGC-3′. *Xenopus* embryonic *ODC* was used for normalization of cDNA samples.

### Western blotting analysis

MOs and mRNAs were injected into four animal blastomeres at the 8-cell stage for dissection of animal caps. Animal caps were dissected at the early gastrula stage (st.10) and were lysed with TNE buffer (10 mm Tris-HCl (pH 7.8), 0.1% NP-40, 150 mm NaCl, 1 mm EDTA, 1 mm DTT and Complete protease inhibitor cocktail (Roche)). The following antibodies were used for Western blotting analysis: Horseradish peroxidase conjugated anti-mouse IgG (GE); anti-SPAK/OSR (Moriguchi *et al*. 2005): anti-p-SPAK/OSR (Moriguchi *et al*. 2005).

### Whole-mount *in situ* hybridization

*In situ* hybridization was carried out using digoxigenin-labeled RNA probe and alkaline phosphatase substrate (NBT) (Roche) according to Harland (1991).
